# ER exit in physiology and disease

**DOI:** 10.3389/fmolb.2024.1352970

**Published:** 2024-01-18

**Authors:** Claire M. Robinson, Aislinn Duggan, Alison Forrester

**Affiliations:** ^1^ School of Medicine, Health Sciences Centre, University College Dublin, Dublin, Ireland; ^2^ Conway Institute of Biomolecular and Biomedical Research, University College Dublin, Dublin, Ireland; ^3^ Research Unit of Cell Biology (URBC), Namur Research Institute for Life Sciences (NARILIS), University of Namur, Namur, Belgium

**Keywords:** secretion, trafficking, endoplasmic reticulum, ER exit sites, COPII, collagen

## Abstract

The biosynthetic secretory pathway is comprised of multiple steps, modifications and interactions that form a highly precise pathway of protein trafficking and secretion, that is essential for eukaryotic life. The general outline of this pathway is understood, however the specific mechanisms are still unclear. In the last 15 years there have been vast advancements in technology that enable us to advance our understanding of this complex and subtle pathway. Therefore, based on the strong foundation of work performed over the last 40 years, we can now build another level of understanding, using the new technologies available. The biosynthetic secretory pathway is a high precision process, that involves a number of tightly regulated steps: Protein folding and quality control, cargo selection for Endoplasmic Reticulum (ER) exit, Golgi trafficking, sorting and secretion. When deregulated it causes severe diseases that here we categorise into three main groups of aberrant secretion: decreased, excess and altered secretion. Each of these categories disrupts organ homeostasis differently, effecting extracellular matrix composition, changing signalling events, or damaging the secretory cells due to aberrant intracellular accumulation of secretory proteins. Diseases of aberrant secretion are very common, but despite this, there are few effective therapies. Here we describe ER exit sites (ERES) as key hubs for regulation of the secretory pathway, protein quality control and an integratory hub for signalling within the cell. This review also describes the challenges that will be faced in developing effective therapies, due to the specificity required of potential drug candidates and the crucial need to respect the fine equilibrium of the pathway. The development of novel tools is moving forward, and we can also use these tools to build our understanding of the acute regulation of ERES and protein trafficking. Here we review ERES regulation in context as a therapeutic strategy.

## 1 Introduction

Protein secretion is an essential and ubiquitous process in eukaryotes. The secretory pathway incorporates biosynthesis, modification and distribution of nascent proteins. Protein secretion is required for all functions of the body, including (but not limited to) providing tensile strength in bones, building connective tissue and skin, forming the extracellular milieu that surrounds cells and provides them with the correct environment to form functioning organs, immunity and cell signalling. Protein biosynthesis and secretion is a complex process that requires dedication of energy and cellular machinery, especially in professional secretory cells, those dedicated to production of large amounts of large proteins, when the biosynthetic pathway becomes one of the cells major occupations. Approximately one-third of proteins in eukaryotic cells are synthesised in the endoplasmic reticulum (ER), where they are modified and folded (reviewed in 1)), packaged and trafficked in COPII-dependent carriers for transport to the ER-to-Golgi Intermediate compartment (ERGIC, reviewed in 2)) then the Golgi apparatus. Post-translational modifications of secretory proteins are made during their transport across different organelles to finalise protein folding in preparation for secretion (Zhang and Wang, 2016). Secretory proteins are guided through the pathway by C and N terminal signal peptides, and a key modification is cleavage of these signal peptides to allow secretion (Zhang and Wang, 2016; Peng et al., 2022). Post-Golgi secretion is determined by characteristics of the cargo itself, with different carriers being employed depending on the characteristics of the cargo; for example, soluble cargo (procollagen) in post-Golgi tubular-saccular carriers (Polishchuk et al., 2003) vs. membrane bound proteins destined for the plasma membrane (E-cadherin) in post-Golgi tubules (Lock et al., 2005).

The biosynthetic pathway is a complex system of vesicular and tubular trafficking. In 2013 the Nobel prize was awarded to Randy Schekman, James Rothman and Thomas Südhof for describing the key mechanisms of vesicle trafficking, thus providing the basis for our understanding of protein trafficking (Balch et al., 1984; Kaiser and Schekman, 1990; Perin et al., 1990; Hata et al., 1993; Söllner et al., 1993; Whittle and Schwartz, 2010). More recently, advances in bioimaging technologies have ignited a new wave of research to unveil the physical details of COPII-dependent carriers, based on the discovery of super resolution imaging. In 2014, Eric Betzig, Stefan W. Hell and William E. Moerner were awarded the Nobel prize for the development of super-resolution fluorescence microscopy (Moerner and Kador, 1989; Hell and Wichmann, 1994; Betzig, 1995; Hell and Kroug, 1995; Dickson et al., 1997; Klar et al., 2000; Betzig et al., 2006), which is now changing the way that we understand protein trafficking, especially at the level of ER exit, which had before remained elusive (Phuyal and Farhan, 2021; Shomron et al., 2021; Weigel et al., 2021).

The ER exit sites (ERES) are a key regulatory step in anterograde protein trafficking. They are areas of the ER deputed to concentrating folded cargo and transporting them to the ERGIC and Golgi, through the orchestration of a set of proteins that regulate and build the COPII coat. This is a highly dynamic process, dependent on transient interactions between the ERES component proteins, and its deregulation can cause catastrophic aberrations in protein secretion as described hereafter. In this review, we use collagen as a model cargo through the different sections, as collagen is central to many pathologies including osteogenesis, cancer and fibrosis, as well as having a complex secretory process. Because of the ubiquitous role of secretion in very common diseases, pharmacological targeting of this pathway is highly sought after. Until recently, specific inhibitors of ERES proteins had not been discovered, but in the last 5 years, two compounds have been identified, Retro-2 (Stechmann et al., 2010; Forrester et al., 2020) and 4-phenylbutyrate (4-PBA) (Ma et al., 2017; Gomez-Navarro et al., 2022a), finally providing proof of principle that ERES can be pharmacologically targeted. Here we discuss the ERES as a key process in the early secretory pathway, its regulation and role in physiology and disease, and perspectives regarding its modulation as a potential therapeutic strategy.

## 2 ERES function

### 2.1 ERES components

Anterograde trafficking and COPII-dependent carriers were originally studied in yeast (Novick et al., 1980; Novick et al., 1981; Newman and Ferro-Novick, 1987; Segev et al., 1988; Kaiser and Schekman, 1990) or reconstituted isolated membranes (Paulik et al., 1988; Beckers et al., 1989; Wilson et al., 1989), describing the proteins involved in vesicular trafficking from ER to Golgi. The hypothesis for the arrangement of the COPII coat was based on electron microscopy of purified inner (Sec23-Sec24) and outer (Sec13-Sec31) COPII coat proteins, determining an icosidodecahedral lattice (Stagg et al., 2008). This model has now been modified in accordance with more recent data (Phuyal and Farhan, 2021; Shomron et al., 2021; Weigel et al., 2021), suggesting the COPII coats can also act as scaffold for transport tubules (Ma and Goldberg, 2016; Hutchings et al., 2018; Hutchings et al., 2021).

Even if the shape of the COPII coat is still under discussion, it is well determined that ERES are a dynamic orchestration of cytosolic proteins that are recruited to the cytosolic side of the ER membrane in turn, where they play three main roles: a) to bend the ER membrane, b) to form a scaffold to maintain membrane deformation and c) to recruit soluble, membrane associated or transmembrane protein cargo. The formation of COPII-dependent carriers is determined by precise cycles of activation - recruitment - deactivation - dissociation of each ERES protein, with exception of Sec12, which does not get recruited or detached. Through dynamic orchestration and transient interactions (Stancheva et al., 2020) the membrane coat is assembled and cargo trafficking achieved. However, the subtle interactions between ERES proteins must be carefully regulated, otherwise COPII cycling becomes deregulated, causing characteristic carrier phenotypes (Van der Verren and Zanetti, 2023).

The initiator of COPII carrier assembly is the ER localised guanine nucleotide exchange factor (GEF) Sec12, that activates GTP-binding protein Sar1, which has two paralogues, Sar1A and Sar1B (Weissman et al., 2001). In its GTP-bound state Sar1 inserts into the ER membrane through its N-terminal amphipathic helix to promote membrane bending and recruits Sec16 and inner coat proteins Sec23-Sec24 (Lee et al., 2005; Long et al., 2010; McMahon et al., 2012). Sec16 defines the ERES in mammalian cells (Connerly et al., 2005; Watson et al., 2006) and it is thought to act as a scaffold for COPII coat formation (Shaywitz et al., 1997; Connerly et al., 2005; Hughes et al., 2009) as well as having a regulatory function on ERES cycling (Bharucha et al., 2013). Sec16 is anchored to the ER membrane via Sec12 (Montegna et al., 2012), and ERES adaptor protein TANGO1 (Maeda et al., 2017). Sec16 binds Sec12, Sec13, Sec23, Sec24 and Sec13, and is hypothesised to stabilise Sar1 activation and the COPII coat (Shaywitz et al., 1997). Not only does Sec16 interact with Sec13 as part of the outer coat, but it forms an essential interaction with Sec13, forming a heterotetramer that stabilises Sec16 (Hughes et al., 2009; Whittle and Schwartz, 2010). Once the inner coat is recruited, the outer COPII coat proteins Sec13-Sec31 are recruited. This heterotetramer is flexible and through binding to Sec23-Sec24 concentrates the inner coat and cargo proteins to form the finalised carrier (Paraan et al., 2018). Importantly, the outer coat heterotetramer also has a regulatory role, stimulating COPII carrier dissociation through stimulation of Sec23 GAP activity (Yoshihisa et al., 1993; Antonny et al., 2001; Bi et al., 2007). This causes Sar1 dissociation from the ER membrane, COPII coat disassembly, and cargo release for anterograde trafficking (Bi et al., 2007; Fromme et al., 2007; Townley et al., 2008).

The classic view of a “standard” COPII vesicle is quoted as being around 60–80 nm. The trafficking of large cargo, such as procollagen fibres of 300–400 nm, thus introduces a problem with the mechanistic hypothesis of carrier formation, requiring that the size must be modulated to accommodate large cargo too (Fromme and Schekman, 2005). Two proteins have been identified as ERES organisers that would resolve this problem, although the question of how large cargo are trafficked, and the organisation of the carrier, are hotly debated topics. TANGO1 (or MIA3) has been shown to recruit ERGIC to the ERES, stabilising and aiding cargo transport, specifically that of collagen (Santos et al., 2015). More recently, TANGO1 has been proposed to create a pH-gradient tunnel or pore for export of collagen from the ER to the ERGIC, avoiding the necessity of the existence of procollagen-suitable megacarriers (Bunel et al., 2024). Returning to a more classical view, TANGO1 is suggested to have roles in COPII stabilisation and cargo recruitment through providing an additional ER anchor for Sec16 (Maeda et al., 2017), recruiting Sec12 and cTAGE5 that is required for collagen ER export (Saito et al., 2014) and secretion (Saito et al., 2011) and also through recruiting Sedlin to the ERES, which interacts with Sar1 to regulate carrier fission, retaining the COPII coat for long enough to allow carrier expansion to accommodate large cargo (Venditti et al., 2012), or indeed to maintain a connecting tunnel between ER and ERGIC to do the same.

**FIGURE 1:**
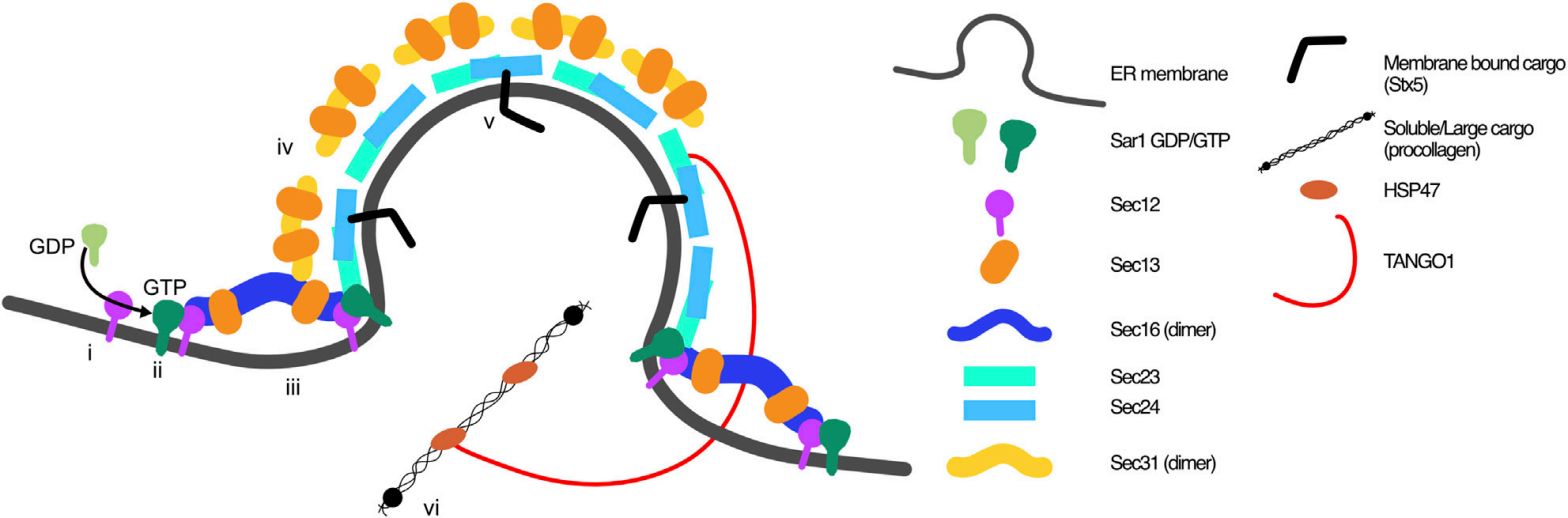
Classic view of COPII coat arrangement at ER exit sites. COPII coats are built through the orchestration of 7 proteins on the cytosolic side of the ER membrane. i) GEF Sec12 activates Sar1, which ii) inserts into the ER membrane and iii) recruits Sec16-Sec13 and inner coat proteins Sec23-Sec24. iv) Outer coat proteins Sec13-Sec31 are recruited to complete the coat. Recruitment of v) membrane bound cargo proteins is aided by cargo receptors (i.e., Sec24), and vi) soluble cargo through ERES proteins including TANGO1, which bind to procollagen via HSP47.

Throughout this review, we will use the well-defined cargo collagen as an example at different stages of the pathway. Procollagens are folded in the ER to form a triple helix from alpha chains, the combination of which depends on which collagen is produced (reviewed in (Ricard-Blum, 2011)). Once folded, procollagen triple helices associate with the collagen-specific chaperone heat shock protein 47 (HSP47), which maintains the triple helical fibral structure, and is a key step of quality control for procollagen folding (Tasab et al., 2000) and regulation of ER export (Ishikawa et al., 2016). The procollagen is subsequently recruited to the ERES and trafficked to the ERGIC then Golgi. This process is dependent on ERES adapter proteins including TANGO1, cTAGE5 and Sedlin, with TANGO1 recruiting HSP47 bound procollagens to load them into transport carriers (Saito et al., 2009; Wilson et al., 2011; Raote et al., 2018; Bunel et al., 2024) (Figure 1). Collagen is one of the most abundant proteins of the body, accounting for 12%–17% total protein in mice (historically this has been estimated at around 30%) (Tarnutzer et al., 2023). Its biosynthesis is complex, and this review will describe how it is commonly implicated in proteinopathies. Thus, here we use it as a model protein to describe the challenges of ER exit, in pathology and disease.

### 2.2 ERES regulation

Biosynthesis and secretion of proteins is an anabolic process that is supported in the cell by a number of regulatory and quality control processes to ensure efficiency of secretion and catabolic balance. RNA interference screens have demonstrated that many proteins can regulate this process (Wendler et al., 2010; Simpson et al., 2012).

The anterograde and retrograde trafficking pathways need to be balanced within the cell and are highly dynamic. In order to efficiently balance and interlink cellular processes, post-translational modifications (PTMs) of all ERES component proteins and some cargo receptors have been reported (Bisnett et al., 2020). These modifications demonstrate the complexity of regulation of the early secretory pathway: the need for fast regulation of ER exit, and the need for repurposing ERES for non-canonical roles, that we have recently started to understand. A long-time hypothesised repurposing of ERES and COPII proteins has been for the biogenesis of autophagosomes (Graef et al., 2013; Karanasios et al., 2016), which has helped to address a long served question of where autophagosome membranes originate. The first recognition of this was in yeast where during starvation, Hrr25 recruitment to COPII components was shown to phosphorylate Sec24, enhancing interaction with Atg9 and driving autophagosome biogenesis (Davis et al., 2016a). Through ULK1 phosphorylation of Sec23B, which inhibits ubiquitin-dependent degradation of Sec23B, and drives a relocalisation of Sec24A, Sec24B and phosphorylated Sec23B to relocate to the ERGIC and promote autophagic flux (Li et al., 2020).

A key auto-regulation mechanism has recently been described: AREX (autoregulation of ER export). Subramanian et al. demonstrate that Sec24 can sense folded cargoes, and as a guanine nucleotide exchange factor, activate ER export and attenuate protein synthesis, thus increasing ER exit on demand. This process flattens out peaks and troughs of protein trafficking, that could be damaging for cell homeostasis (Subramanian et al., 2019).

Tillman et al. showed that through MAPK signalling, the early secretory pathway is linked to nutrient deprivation, integrating growth factor signalling with cell proliferation through Sec16 (Tillmann et al., 2015), as well as the major catabolic process, autophagy (Zacharogianni et al., 2011). Not only is it logical that autophagy is regulated along-side secretion to balance energy expenditure with use, but formation of autophagosomes also requires donor membranes. The ER and ERGIC (Ge et al., 2014a; Ge et al., 2014b) are considered to be key donors and sites of omegasome formation (phosphatidylinositol 3-kinase (PI3K) positive membranes that precede nascent autophagosomes) (Axe et al., 2008; Hayashi-Nishino et al., 2009). Mechanistically, it has been shown that the cargo adaptor and Sec12-binding protein cTAGE5 as well as ER-localised FIP200 modulate autophagosome biogenesis through ERES remodelling (Ge et al., 2017). In yeast that lack the ERGIC, phosphorylation of Sec24 has been shown to increase its interaction with essential autophagy protein Atg9, to increase abundance of autophagosomes during nutrient deprivation (Davis et al., 2016b).

More recently, alternative splicing has been described as a Sec16-dependent mechanism for the fast regulation of COPII transport, in adaptive responses such as increased cargo load during T cell activation (Wilhelmi et al., 2016), balancing COPII cycling with autophagy in erythroid differentiation (Cendrowski et al., 2020), and to protect against mechanical stress via small GTPase Rac1, a regulator of actin cytoskeleton remodelling, which modulates ERES number and rate of ER exit through interacting with small GTPase Sar1 (Phuyal et al., 2022).

The ERES is tightly controlled by a number of processes within the cell, demonstrating its pivotal role in the integration of cellular signalling, and maintenance of the equilibrium of anabolic and catabolic processes within the cell. Thus, targeting ERES pharmaceutically may have wider effects on cellular homeostasis, but it also provides the opportunity to use ERES modulation to re-equilibrate the cell when that balance is lost.

### 2.3 ERES inhibition

Although many physiological mechanisms and pathways have been shown to regulate ER exit, very few mechanisms of specific pharmacological intervention have been identified. The tools used to study ERES modulation in the lab are classic, but flawed: silencing RNA is commonly used to remove ERES component proteins, inhibiting ERES formation (Sec16A) (Watson et al., 2006), or trafficking (Sar1A/B) (Melville et al., 2020), but these approaches take days to deplete proteins, after which time the highly dynamic ER and ERES can compensate for the modulation, and if they do not, the global disruption to trafficking may become lethal. Using a model of chronic disruption does not allow us to see the subtle consequences that would allow us to truly understand the distinct roles of ERES proteins, and especially not the acute consequences of ERES inhibition. Compounds are widely used to inhibit trafficking, such as Brefeldin A, nocodazole, and monensin, but these compounds effect total trafficking and disturb the homeostasis of the whole cell (Pelham, 1991; Badr et al., 2007; Aowicki and Huczyński, 2013). To date, very few compounds have been identified that specifically target ERES, although compounds that target the secretory pathway have been identified (Zhao et al., 2018). The first compound identified as specifically targeting the ERES was Retro-2 which was first discovered as an inhibitor of retrograde Shiga toxin trafficking through targeting Sec16A (Lee et al., 2005). This compound inhibits anterograde trafficking of Syntaxin5, decreasing its interaction with GOLIM4, which is required for endosome to Golgi trafficking of Shiga toxin (Stechmann et al., 2010; Forrester et al., 2020). The second ERES targeting compound identified was 4-PBA, a known regulator of transcription and ammonia scavenger that has been used clinically since the 1990s (Chen et al., 1997). In 2017 Ma et al. identified its effect on reversing ER retention of misfolded proteins. The drug was shown to block secretion of PCSK9 through inhibiting the interaction between its cargo receptor SURF4 and Sec24A (Ma et al., 2017; Gomez-Navarro et al., 2022b). As described in this review, ERES are regulatory hubs and play a crucial role in balancing cellular homeostasis. However due to the high number of common diseases that are linked to ERES disfunction (see Section 4), the identification of compounds that could modulate ERES would be incredibly valuable. Both of these compounds were identified serendipitously. But they provide proof of concept that ERES proteins can be targeted pharmacologically. Paired with recent improvements in technologies that provide us with approaches of increased sensitivity and resolution, we can now search directly for inhibitors that specifically target ERES proteins and that can be finely tuned to inhibit trafficking of an individual protein to a specific degree or at a defined trafficking stage, to maximise therapeutic outcomes over off-target effects.

## 3 ERES quality control, feedback and ER stress

The ER processes approximately 35% of the proteome (Braakman and Hebert, 2013). The process of folding and modifying large numbers of nascent proteins is challenging for a cell, and emergence of misfolded proteins in the ER is a continuous event that must be tightly controlled to maintain cellular homeostasis (Schubert et al., 2000). To ensure that dysfunctional proteins are not released into the secretory pathway, ER chaperones act to retain unfolded proteins until they can be further modified, but failing that, unfolded proteins must be removed. As such, the ER senses misfolded proteins (reviewed in (Wiseman et al., 2022)). Quality control mechanisms then direct the delivery of proteins that do not meet the conformational standards to the cytosolic protein degradation machinery via ER associated degradation (ERAD) (Carvalho et al., 2006) or to lysosomes via autophagy (ER-phagy and ERLAD, reviewed in (Reggiori and Molinari, 2022)). Together, quality control chaperones promote folding, and degradation pathways remove misfolded proteins, to certify that only properly folded proteins exit the ER and continue in the secretory pathway. In addition, these mechanisms can also contribute to adaptive responses during stressful or pathological scenarios (Fregno et al., 2018).

### 3.1 ER associated degradation (ERAD)

Removal of proteins from the ER lumen can occur not just by exit, but also by degradation. Selected proteins in the lumen of the ER are retrotranslocated across the ER membrane to the proteasomal machinery for their degradation via a protective mechanism termed ERAD (Needham and Brodsky, 2013). Although misfolded proteins have been the most infamous target for ERAD, native proteins, and thus their linked processes, can also be regulated (reviewed in (Kumari and Brodsky, 2021)). Inactivation of ERAD leads to the accumulation of proteins in the ER lumen, deregulating pathways dependent on native ERAD substrates, and most importantly in the context of this review, misfolded proteins that can lead to diseases including proteinopathies (Reviewed in (Krshnan et al., 2022)). These include nephrin (retention in the ER of mutants can cause hereditary nephrotic syndrome, Online Mendelian Inheritance in Man [OMIM]: 600,995 (Huber et al., 2003; Yoshida et al., 2021), proinsulin (He et al., 2015; Hoelen et al., 2015) and α1-antitrypsin Z (ATZ) (Wang et al., 2011). Unsurprisingly, inactivation of a selection of genes involved in ERAD leads to embryonic lethality in mice, indicating the importance of ERAD in cellular homeostasis (Yagishita et al., 2005; Francisco et al., 2010).

ERAD machinery must be able to distinguish between misfolded proteins *versus* proteins that are undergoing the maturation process. This is generally orchestrated by molecular chaperones, including BiP, calnexin (CNX) and calreticulin (CRT), that interact with unfolded proteins (reviewed in (Vembar and Brodsky, 2008)). The location of the folding defect on the protein can influence the ERAD machinery used to instruct destruction (Carvalho et al., 2006). Another recognition mechanism that is common for secretory proteins is glycosylation. Nascent proteins are glycosylated upon translocation into the ER lumen. The glycan chain is then modified through glucosidase I and II activity, producing a monoglycosylated protein that is recognised by ER chaperones: membrane bound CNX and soluble CRT (Kozlov et al., 2010). This is termed the CNX-CRT cycle, retaining proteins to undergo multiple cycles of modification, which continues until complete folding is achieved, signalled by removal of the last glucose. Complete folding is monitored by UDP-glucose: glycoprotein glucosyltransferase, which can reglycosylate proteins that do not achieve folding on the first round to guide them to re-enter the CNX-CRT cycle until folding is achieved (Soldà et al., 2007), and they can be released for ER exit. However, if 3-4 mannose residues (of the original 7 in Glc3Man9 GLcNAc2) are removed from the oligosaccaride chain, this indicates that proteins are irreversibly misfolded. When misfolded proteins are recognised, they are removed from the ER, and polyubiquitinated by ubiquitin ligases that leads to destruction by the proteasome (Needham and Brodsky, 2013).

ERAD machinery is concentrated within specific regions of the ER, termed ER-derived quality control (ERQC) compartments. It has been suggested that in some cases, the COPII machinery is required in order to have fully functional ERAD, whereby misfolded proteins use COPII-dependent transport to access the ERQC compartment (Kakoi et al., 2013; Ogen-Shtern et al., 2023). Earlier studies reveal that sequestration of the cystic fibrosis transmembrane conductance regulator (CFTR) into ERAD is disrupted when Sec12, Sec13 or Sec24 are mutated in yeast (Fu and Sztul, 2003). A similar reliance on COPII components has been reported in mammalian cells (Ogen-Shtern et al., 2023).

Given these highly reactive quality control processes, it seems likely that ERES inhibition could result in re-direction of proteins to ERAD, but this interaction remains to be demonstrated.

### 3.2 Targeted ER degradation

Synthesis of large proteins such as collagen comes with additional complexity and challenges for the ER. Some large proteins are resistant to proteasomal degradation, as their size inhibits them from passing through retrotranslocation machinery used by ERAD (Hamman et al., 1997), meaning that without an alternative route of degradation, they would accumulate within the ER with toxic consequences. Recently, alternative degradation pathways have been well described, that utilise the canonical cellular degradation machinery: the lysosome (Khaminets et al., 2015; Fumagalli et al., 2016; Grumati et al., 2017; Fregno et al., 2018; Forrester et al., 2019; Sun et al., 2023). Autophagy was initially shown to be required during long bone growth, followed by its identification as a quality control pathway in ER-phagy (Cinque et al., 2015; Forrester et al., 2019). It plays a key role in the maintenance of homeostasis in the anterograde trafficking pathway, specifically in the trafficking of endogenous type I and II procollagen during post-natal bone growth, a role especially important during periods of high secretory workload. Three distinct pathways have now been described: ER-phagy, ERLAD and recently, ERES-phagy.

ER-phagy is a selective autophagy pathway that uses ER-specific autophagy receptors to regulate turnover or remodelling of ER membranes or to clear “trouble” (excessive or misfolded) cargo. Turnover of the ER maintains homeostasis and ER function, using ER-phagy receptors specific to ER-subdomains; FAM134B for ER sheets (Khaminets et al., 2015) and reticulon-3 for ER tubules (Grumati et al., 2017). Remodelling of ER after enlargement induced by ER stress (see Section 3.3) utilises Sec62 (Fumagalli et al., 2016). However, the role most interlinked with ERES function is the degradation of ER proteins which need to be removed due to misfolding or overloading of the ER compartment, the latter of which can be caused by increased protein biosynthesis (e.g., during times of high secretory workload) or decreased ER exit. It remains to be shown whether blockage in the secretory pathway can regulate ER-phagy, however it is known that during starvation, lysosomes can downregulate translation through the mTOR - transcription factor EB (TFEB) and E3 (TFE3) pathway (Laplante and Sabatini, 2013), and that this induces ER-phagy through inducing expression of ER-phagy receptor FAM134B (Cinque et al., 2020).

The number of endogenous ER cargoes recognised to be degraded by ER-phagy is increasing, and with it, potential links to disease that are tempting therapeutic targets. The first involvement of autophagy in quality control of cargo trafficking was demonstrated during periods of high cargo load: type II procollagen secretion during postnatal bone growth. This work linked aberrant signalling in achondroplasia to deregulation of autophagy, which decreased type II procollagen secretion (Cinque et al., 2015). The role of autophagy in this system was then shown to be ER-phagy, via a mechanism that exploits the binding of unfolded type I or II procollagen to ER chaperone calnexin, which interacts with ER-phagy receptor FAM134B (Cinque et al., 2015; Forrester et al., 2019). ER-transmembrane protein Niemann-Pick type C (NP1) is degraded in a FAM134B dependent manner, and recently phosphatidylinositol-3-phosphate (PI3P)-binding protein TOLLIP has been identified to promote degradation of mutated ER transmembrane proteins including spastic paraplegia-associated Seipin and an amyotrophic lateral sclerosis (ALS)-linked mutant of VAPB (Hayashi et al., 2023). Aggregated proteins have been a favourite target for therapies aimed at clearing problem proteins from the ER. ATZ aggregates in the ER and is degraded in the lysosome, which was shown to be an alternative route of ER-degradation, ERLAD (ER-to-lysosome-associated degradation) which is notably not regulated by the mTOR pathway (Fregno et al., 2018). The same group has also now demonstrated that blockage of ERAD re-routes proteins for degradation by FAM134B-dependent ERLAD routes, describing a novel compensatory link between ERQC pathways (Fasana et al., 2023).

Finally, an ERES specific, quality control role for autophagy has been described, revealing the switch between secretory ERES to degradative ERES. Caused by the accumulation of proteasome-resistant ATZ at the ERES, COPII trafficking is disrupted, causing an accumulation of ERES localised proteins, most importantly the membrane embedded V0 subunit of the V-ATPase. Although this is found at the ERES during steady state, the accumulation recruits the soluble V1 subunit, and eventually breaches a threshold triggering recruitment of ATG16L1-ATG5-ATG12, then via FAM134B-II, LC3C is recruited to complete the ERES-phagy complex (Sun et al., 2023).

Acute responses to changes in the secretory system can therefore trigger compensatory mechanisms, interlinking ERQC and protein homeostasis to trafficking. ERES modulation provides a tool with which we can understand the acute responses to modulation of the secretory pathway, and use ERES modulation as a mode to regulate interlinked homeostatic processes.

### 3.3 ER stress

Internal and external insults can elicit a stress response in the ER as a consequence of accumulation of misfolded proteins. Internal perturbations include mutant protein expression or situations where there is a significantly increased demand for cargo. External stimuli include hypoxia; oxidative stress; significant changes in calcium levels or localisation; or nutrient deprivation (Koumenis et al., 2002; Koritzinsky et al., 2006; Malhotra and Kaufman, 2007; Groenendyk et al., 2021). To minimise or overcome ER stress, cells respond through activation of an adaptive response called the unfolded protein response (UPR). The UPR comprises 3 transmembrane proteins: Inositol-requiring enzyme 1 [IRE1]; Protein kinase R (PKR)-like ER kinase [PERK]; and activating transcription factor-6 [ATF6], that together initiate transcriptional and translational responses that increase the capacity of the ER for protein folding, reduce global translation and accelerate protein degradation (Walter and Ron, 2011). Together, these activities aim to restore protein balance within the ER. However, if the stress is too great to overcome and ER balance cannot be restored, the UPR can be pro-apoptotic (Tabas and Ron, 2011).

In unstressed conditions, inactive transcription factor ATF6 binds to BiP at the ER. Upon ER stress, it dissociates from BiP and undergoes COPII-dependent transport to the Golgi where it is activated by undergoing cleavage by site-1 and site-2-proteases (S1P and S2P) (Nadanaka et al., 2004; Schindler and Schekman, 2009). It has been shown that ATF6 is one of a specifically selected set of proteins that are transported to the ERGIC and Golgi in ER stress scenarios (Nadanaka et al., 2004). Significant ER stress has also been reported to cause ER whorls. Formation of these whorls are thought to be a PERK dependent phenomenon and rely on COPII components to elicit their formation (Xu et al., 2021).

The interaction between ER stress and ERES is complex, and is likely to depend on the model system, environment and cargo, in some cases increasing anterograde trafficking (Farhan et al., 2008), in some cases decreasing it (Amodio et al., 2013). One of the main factors causing an increase in ERES cycling (thus anterograde trafficking) is high cargo density, an auto-response to maintain homeostasis (Farhan et al., 2008; Subramanian et al., 2019). There is also evidence for direct regulation of ERES by the UPR. A 2019 study found that COPII-mediated trafficking mirrored the activation of IRE1, whereby activation of the IRE1-XBP1s axis led to increased ER-Golgi trafficking in the liver. The same study performed XBP1s ChIP-seq and mRNA quantification to demonstrate direct XBP1s mediated transcription of ERES components Sec23B and Sec16A (Liu et al., 2019). There is limited evidence suggesting that ATF6, independent of XBP1s, is capable of inducing expression of secretory pathway components, including ERES components, although this requires confirmation (Bommiasamy et al., 2009). Recently, it has been shown that in mouse fibrosis models, genetic depletion of IRE1 reduced liver damage and decreased collagen deposition (Hazari et al., 2023). This suggests that an ERES modulating drug could also be used to alleviate ER stress and the diseases related to it, however this hypothesis would need to be studied in cargo and model specific situations. Acute modulation of ERES will however provide an interesting tool to study the fast cellular reactions to secretory alterations.

## 4 ERES in disease

**FIGURE 2:**
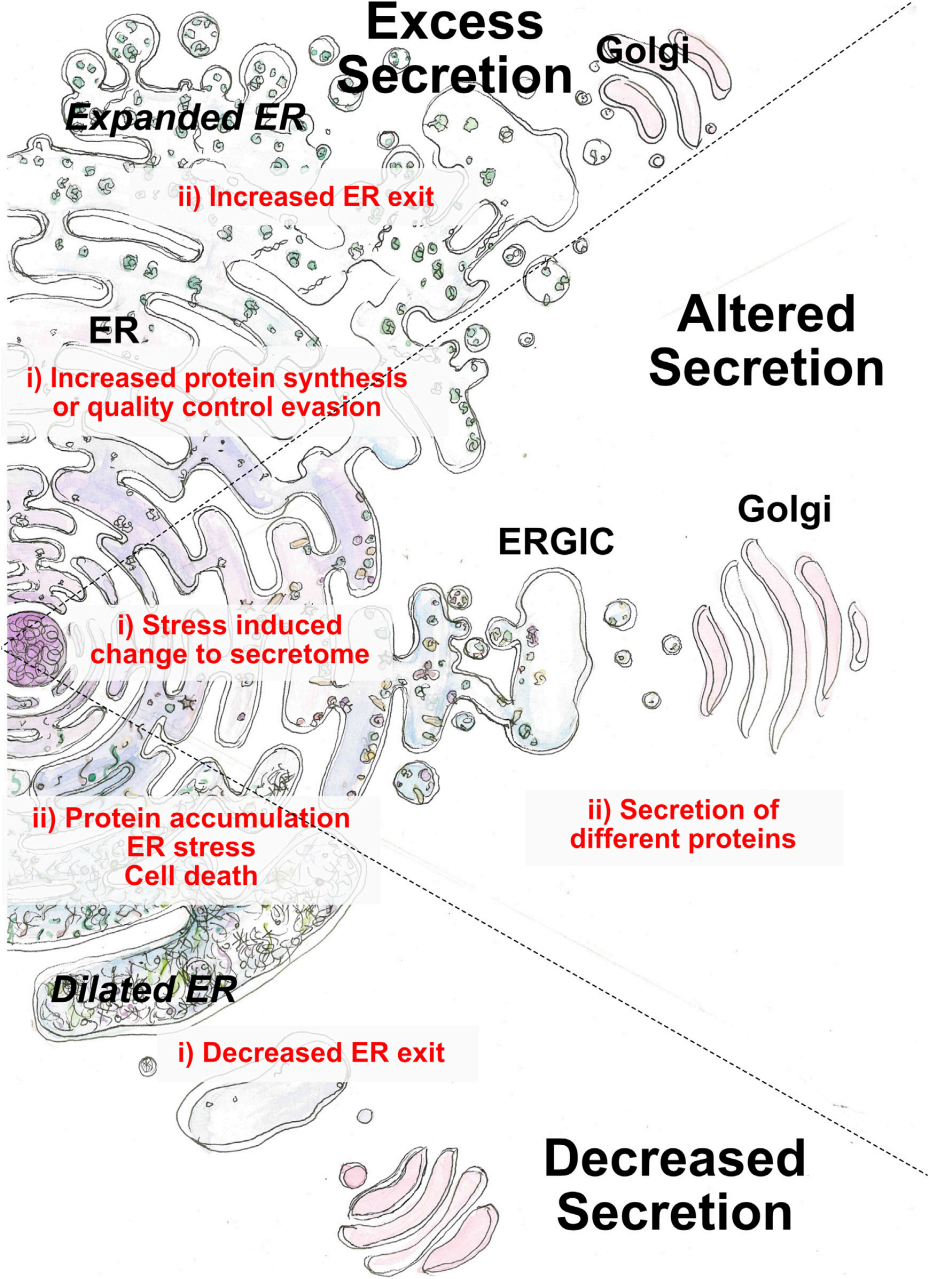
Perturbation of the early secretory pathway in disease states. Three main categories of aberrant secretion can lead to disease: decreased, excess or alternative secretion. Causative factors (i) originate from cargo or ER exit. Outcomes (ii) arise from loss of cellular homeostasis or loss of physiological function regarding cargo function.

A good source of information on the consequences of ERES inhibition is to look at the natural effects of ERES component mutations. Considering the ubiquitous role of secretion throughout the body, there are surprisingly few groups of diseases directly related to ERES mutations. Here we describe them related to their cause, starting with the most informative: *ERES component mutations*, followed by an example of a common disease of excess extracellular matrix (ECM) secretion (fibrosis), description of the role of an altered secretome in cancer, and finally the consequences of decreased secretion (Figure 2). It is evident that many COPII-dependent diseases are related to aberrant ECM secretion, especially that of our model cargo collagen. It is also worth noting that some proteins are absent from this list (i.e., Sec16), as no disease-causing mutations have been identified in them to date. In some cases, mutations in these proteins can be embryonically lethal, and it is interesting to consider whether this would account for the majority of ERES component mutations.

### 4.1 ERES component mutations

Based on the discovery of broad inhibitors of trafficking, and the research performed over the last 40 years, it has been presumed that inhibiting ERES would cause broad inhibition of protein trafficking. However, looking at the consequences of these mutations, we see examples of collagen-specific inhibition and very specific pathologies. This suggests that COPII is highly specific also *in vivo* and we *can* consider specific inhibitors as a viable strategy for collagenopathies. For example, one of the most well defined COPII-related diseases is cranio-lenticulo-sutural dysplasia (CLSD, OMIM: 607812), that was first linked to mutations in Sec23A (see below) (Boyadjiev et al., 2006). This association has provided insights into the function of COPII components, especially the necessity for efficient coupling of Sec23-Sec24 to Sec13-Sec31, to drive ER exit of procollagen, that is essential for normal craniofacial development (Townley et al., 2008). It also provides other interesting perspectives, as it is clear that for such a key pathway, the range of diseases robustly linked to mutations in the ERES components is relatively narrow.

Sar1B Mutations in SAR1B were identified in a number of families with chylomicron retention disease (OMIM: 246700) (Jones et al., 2003; Charcosset et al., 2008). In most cases, the mutations identified resulted in a decreased affinity of SAR1B for GDP/GTP, thus becoming unable to be activated. An additional patient was found to have a mutation that truncated SAR1B. This caused a defect in assembly of chylomicrons due to disrupted glycosylation, causing severe fat malabsorption (Levy et al., 1987).

Sec23A Mutations in Sec23A were found in a number of patients with CLSD (Boyadjiev et al., 2003; Boyadjiev et al., 2011). Although the mutation does not affect binding capabilities of Sec23A, the GAP activity stimulated by Sec13-Sec31 was very limited. In patient fibroblasts, this resulted in less vesicle budding, retention of protein in the ER and consequently gross dilation (Boyadjiev et al., 2006), and accumulation of cargo-containing tubular structures, devoid of COPII coats (Fromme et al., 2007). This data led to the description of two stages for COPII coat formation: the pre-budding complex Sar1-Sec23-Sec24 that is sufficient to build cargo-containing tubules, and the Sec13-Sec31 complex that is required for membrane fission, or the final steps of COPII-dependent transport (Fromme et al., 2007).

Sec23B Mutations in Sec23B have a number of different disease phenotypes. The first is congenital dyserythropoietic anaemia type 2 (CDAN2, OMIM: 224100), where mutations destabilise Sec23B protein and disrupt glycosylation of transmembrane proteins in red blood cells (Bianchi et al., 2009; Schwarz et al., 2009). Mutations in Sec23B have also been linked to oncogenic phenotypes and are found in cancer (discussed in Section 4.3). In Cowden syndrome (OMIM: 616858), a missense mutation in Sec23B was identified in patients that inhibited binding to Sar1 and caused Sec23B aggregation, inducing a cancer-like phenotype in HEK293T cells (Yehia et al., 2015).

Sec24D Cole-Carpenter Syndrome type II (CLCRP2, OMIM: 616294) is an osteogenesis imperfecta-like syndrome, with skeletal dysplasia and low bone mass (Takeyari et al., 2018). Although mutations have been identified throughout the Sec24D gene, interestingly some lie within the gelsolin-like domain, a domain that has been shown to be responsible for transient interactions between ERES proteins (Stancheva et al., 2020). A general phenotype of the CLCRP2 mutations is to cause inefficient ER exit of procollagen, and significant dilation of the ER (Garbes et al., 2015).

Sec31A A loss of function mutation has been linked to Halperin-Birk syndrome in one family (OMIM: 618651). The syndrome describes a severe neurodevelopmental disorder with wide ranging developmental (including skeletal) defects (Halperin et al., 2019). The disease was phenocopied in Sec31A null *Drosophila* and Sec31A deficient SH-SY5Y and HEK293T cells. *Drosophila* showed brain defects and early lethality, while cell models showed increased ER-stress-dependent cell death (protein trafficking was not studied) (Halperin et al., 2019).

### 4.2 ERES organisation proteins

Although not part of the COPII coat, TANGO1 and SEDLIN are key modulators of ERES and cargo trafficking from ER to ERGIC. Although their precise functions are still poorly characterised, their importance is underlined by the consequences of their mutations, and thus their potential as therapeutic targets.

TANGO1 Total loss of TANGO1 is embryonically lethal in humans, causing a nearly total absence of mineralised bone, which is phenocopied in TANGO1 null mice, although in humans, heterozygous parents are healthy (Guillemyn et al., 2021). A TANGO1 truncation mutation was identified in patients with a novel syndrome displaying dentinogenesis imperfecta, short stature and skeletal abnormalities, insulin-dependent diabetes mellitus, sensorineural hearing loss and mild intellectual disability. *In vitro*, this mutation was shown to cause dispersal of TANGO1 through the ER, and to decrease type I collagen secretion (Lekszas et al., 2020). TANGO1 is from the *Melanoma Inhibitory Activity* gene family, named after the discovery that MIA1 expression inversely correlates with melanoma progression (Bosserhoff et al., 1997). Subsequently, MIA2 and MIA3 (TANGO1) were discovered, and although there is little evidence linking them to melanoma, they have been shown to be involved in other cancers: overexpressed TANGO1 provides a link to oncogenesis through promoted expression of CHAC1 and degradation of glutathione (GSH), thereby promoting the growth and metastasis of hepatocellular carcinoma cells, while its knockdown blocks tumour growth and metastasis (Wanbiao et al., 2023).

Sedlin The Sedlin protein was named after the disease that brought about its discovery, spondyloepiphyseal dysplasia tarda (SEDT). It was shown initially to interact with membrane regulatory chloride intracellular channel proteins (CLIC) 1 and 2, that regulate homeostasis of membrane potential in organelles, cell size and intracellular pH (Fan et al., 2003). However, the key role here for Sedlin is as an ERES organiser for large cargo. Sedlin regulates the membrane recruitment cycle of Sar1 through binding to it, and thus modulates the cycling speed of COPII carrier formation. Mutations found in SEDT patients increase the time that Sar1 remains at the membrane, inducing constriction and early fission of nascent carriers, thus restricting the size of the COPII carriers, and the size of the cargo that can be transported. Sedlin is required for efficient ER exit of large cargo proteins, while small cargoes are not sedlin-dependent (Venditti et al., 2012). This mechanism is supported by the known involvement of mutations in type II procollagen that can also cause SEDT (Rolvien et al., 2020).

Specific mutations of ERES proteins provide *in vivo* data that identify the consequences of targeting these proteins pharmacologically. Given their exquisite specificity, it provides a template to allow us to study drug targets, and to identify which drugs could be used to achieve which consequences in drug development studies.

### 4.3 Fibrosis

Fibrosis is characterised by excessive accumulation of ECM components in a tissue (Herrera et al., 2018), that is caused by a loss in balance between production and degradation (Zhao et al., 2022). Fibrosis is a consequence of chronic inflammation that can arise from injury, but it can also be idiopathic in nature, for example, in the case of idiopathic pulmonary fibrosis. The accumulation of fibrotic “scar” tissue ultimately leads to deterioration of organ function (Wynn and Ramalingam, 2012). At the crux of the fibrotic pathology are myofibroblasts that produce excessive matrix components including vast quantities of collagens, one of the main structural components of the ECM. Cytokines and other soluble factors secreted from myofibroblasts, as well as other cell types in the diseased organ, can promote disease progression by creating a pro-fibrotic milieu in the extracellular space (Kendall and Feghali-Bostwick, 2014).

Myofibroblasts can be derived from many sources including fibroblasts, stellate cells and smooth muscle cells (Hinz et al., 2007). Notably, myofibroblast activation is accompanied by stress-induced enlargement of the ER, indicative of increased secretory burden (Koo et al., 2016). To accommodate this, there are also changes in the expression of COPII coat proteins: in a study in idiopathic pulmonary fibrosis, Sec24A gene expression was significantly upregulated in diseased tissue relative to healthy tissue controls (Ghandikota et al., 2022). From a therapeutic standpoint, interfering with the secretory pathway of myofibroblasts could be a worthwhile approach to treat this group of diseases (Staab-Weijnitz, 2022).

Collagens are one of the main secreted components of fibrosis-generating cells. In activated hepatic stellate cells, TANGO1 depletion decreases the level of secreted type I collagen. In the same study, two separate murine TANGO1 ± models of hepatic fibrosis were tested and a decrease in disease severity was observed in both, reinforcing the importance of TANGO1 at ERES (Maiers et al., 2017). While it is encouraging to observe modulation of collagen transport at ERES, there remains much to learn about these approaches and the notion of modulating TANGO1, or other collagen regulatory proteins, in myofibroblasts.

As well as the structural ECM contribution to fibrosis, there is also mechanistic involvement of cytokine, chemokine and growth factor secretion. While some cytokines, e.g., IL-15, IL-1b do not enter the secretory pathway, many others such as IL-2, IL-6, IL-10 and TNF contain a signal peptide targeting them to the ER where they are correctly folded and recruited for COPII-dependent transport. Both innate and adaptive immune cells as well as myofibroblasts can contribute to a secretome that contains a mixture of pro- and anti-fibrotic factors (Zhang and Zhang, 2020). Unsurprisingly, ERES are important in the transport of many of these factors from the ER to the Golgi. A study in T cells demonstrated that the COPII pathway is utilised to deliver secreted factors upon T cell activation. In human and murine T cells both Sec23A and Sec23B are expressed, Sec23B to higher levels. Interestingly, loss of Sec23B led to perturbation of secreted effector cytokines, including IL-2, IFNɣ, IL-4, IL-9 and TNF while having no effect on secretion of others such as IL-10 and CCL4 (Kim et al., 2021). Other factors involved in fibrotic promotion include CXCR4 and angiotensin II which have also been identified as COPII-dependent cargo, further supporting the notion that ERES machinery may be therapeutic targets in fibrotic diseases (Xu et al., 2023).

### 4.4 Cancer

Oncogenesis, the process by which normal cells become cancer cells, is associated with aberrant changes in secretion which, via autocrine and paracrine signalling can positively impact tumour progression. For example, acquisition of p53 mutation is widely reported to alter the cancer cell secretome (Cordani et al., 2016). Cancer-associated secreted factors promote a tumour microenvironment that drives disease progression via multiple mechanisms, including resistance to therapy, enhancing survival in harsh conditions and promoting immunosuppression to evade the body’s defenses. As such, modulation of the cancer secretome is an attractive avenue of therapeutic intervention.

There is clear evidence that oncogenic transformation impacts ERES. For example, RAS, the most commonly mutated gene in human cancers, can control the number of ERES via regulation of ERK2. When oncogenic RAS (RasV12) is expressed, ERK2 phosphorylates Sec16 that leads to increased numbers of ERES (Farhan et al., 2010). Indeed, Sec16 has been proposed as a master regulator or integrator of growth factor signalling. Growth factors regulate Sec16A levels which has an impact on ERES and secretion. This may explain why decreased expression of Sec16A decreases proliferation while increased expression has the opposite effect (Tillmann et al., 2015).

The ERES machinery has been reported to be mutated or changed in some cancers. A study of common mutations in sporadic cancers using the Cancer Genome Atlas, identified that sporadic thyroid cancer had the highest incidence (4%) of Sec23B mutations, compared to invasive breast cancer and uterine corpus endometrioid cancer. On average, the median age of cancer onset was significantly lower in patients harbouring a Sec23B mutation than those with wild type Sec23B (36 vs. 46 years old). Additionally, 75% of sporadic cancers in the database showed overexpression of Sec23B transcripts, with 10% showing a high level of transcript amplification, suggesting that the mutations are likely to be deleterious. This demonstrates that Sec23B mutations have a role in sporadic cancer formation, suggesting a more important role for Sec23B in carcinoma than is currently understood. Sec24B has also been assessed in multiple cancer types where it was found to be highly expressed relative to normal tissue. In oesophageal carcinoma, lung adenocarcinoma and kidney renal papillary cell carcinoma, higher Sec24B expression correlated with shorter survival (Du et al., 2023). Modulating Sec23B levels using miR200, has also been implicated in metastasis whereby decreases in Sec23B decreased metastatic behaviours in cells (Korpal et al., 2011). Cargo receptors and adapters are also perturbed in some cancers. For example, microsatellite unstable colorectal cancer (CRC) is characterised by an aberrant glycosylation profile and a high mutational frequency of ERGIC53, a carrier that assists ER to Golgi transport (Roeckel et al., 2009). Elsewhere, SURF4, the cargo receptor for erythropoietin, has also been investigated in cancer models where its expression is high in breast cancer and is associated with increased proliferation and migration of NIH3T3 and tumour cells (Kim et al., 2018; Lin et al., 2020; Zhai et al., 2022).

Unsurprisingly, given high frequency of mutations and a high rate of proliferation and metabolic demand, ER stress and activation of the UPR are commonly observed in cancer. Specifically, the IRE1-XBP1s arm is often constitutively active in cancer cells from many tumour types including breast, brain and haematological malignancies (Lhomond et al., 2018; Logue et al., 2018; Robinson et al., 2021; Crowley et al., 2023). This constitutive activation has been implicated in multiple oncogenic signalling activities and intense efforts are ongoing to target the IRE1-XBP1 axis in cancer. RNase and kinase specific inhibitors of IRE1 have been developed, one of which (ORIN001) has entered clinical trials for the treatment of metastatic cancer (https://clinicaltrials.gov/study/NCT03950570). Notably, inhibition of IRE1 in cancer models leads to the modulation of the tumour secretome (Logue et al., 2018; Creedican et al., 2022). As expected, *bona fide* XBP1s target genes are impacted (e.g., IL-6), but the modified secretome also encompasses other factors that are not under direct control of the IRE1-XBP1s axis and may be a consequence of IRE1 mediated ERES regulation (Liu et al., 2019).

This remains to be investigated. This tight association of pro-oncogenic environment and altered secretome, as well as the contribution of ER-stress to cancer development, thus suggests that drugs able to modulate ERES may have a positive effect on ER-stress involvement or in more specific cases, the normalisation of the cancer cell secretome, in development of the pro-tumour environment.

## 5 Discussion

Anterograde trafficking is a ubiquitous and essential pathway, regulated and regulating many varied processes within the cell. This review outlines the importance of efficient ER exit, and the related processes that are in place to maintain homeostasis of the organelle, cell and tissue, from the perspective of pharmacological modulation of ERES to address diseases of aberrant secretion.

From the high frequency of diseases that are caused by aberrant secretion, maintaining equilibrium in the secretory pathway is apparently crucial to homeostasis at the level of the cell, organ and organism. Although diseases of aberrant secretion are very common, few effective therapies exist, even for the most common diseases, such as fibrosis. Cancer and fibrotic diseases are characterised by significant changes in the secretory pathway, including scenarios of increased secretion and modification of cargo. While efforts have been made to target aspects of the secretory pathway in these diseases, ERES specific targeting has yet to be explored. Whether this approach can modify secretory pathway proteins enough in pathogenic cells whilst retaining function in non-diseased cells remains to be determined. The incidence in disease states of overexpression or mutation of COPII coat components would suggest that selective targeting of these proteins may be possible. The challenge to develop these pharmaceuticals will be to inhibit targeted proteins to a certain level in a reversible manner, to avoid inducing a catastrophic inhibition of protein secretion that will mimic the diseases described in Section 4.

The number of disease-linked ERES component mutations is surprisingly small, and the range of diseases notably skewed towards collagenopathies and ECM related disease. This may suggest that many mutations in the other ERES component proteins are embryonically lethal (as there are already some examples). It is also interesting to see that in many ERES proteins, disease-linked mutations are spread throughout the gene, rather than clustered in one location. This supports that the role of each ERES protein is complex: interacting with multiple proteins, maintaining enzymatic activity and structure for proper localisation and recruitment.

In mammals, all ERES proteins except for Sec12 (thus all ERES proteins recruited to the cytosolic side of the ER), have multiple paralogues, although well-defined roles for these paralogues have not been robustly shown. This raises an interesting evolutionary question: why are so many paralogues needed? From an evolutionary perspective, redundant paralogues are unfavourable, as evolutionary pressure would not maintain the multiple proteins’ functions. It could be more likely that they serve as a threshold, to provide the high (and tuneable) levels of protein required to maintain the high number and level of activation of ERES. It is beneficial for the cell to maintain multiple copies of different proteins, than to express one protein to very high levels (Thompson et al., 2016). Looking at the divergence of the paralogues would allow us to build a hypothesis around this theory.

With the emergence of compounds that can modulate ER exit, we can start to understand the rapid feedback pathways that exist within the secretory pathway, and thus the complexity of modulating distinct processes within it. Thanks to the multi-functionization of some COPII components by post-translational modification to play non-ERES roles, the chances of ERES inhibition having effects on interlinked processes is high. Interestingly, some of these connections will not be recognised with the tools that we currently have (genetic intervention), that modulate the ERES slowly as they work on genetic ablation and degradation of COPII proteins. This slow change allows the early secretory pathway, which is especially dynamic, to compensate for changes in ER exit, maintaining organelle homeostasis. However, with new tools that are available to us, we may start to uncover new links between processes that respond rapidly to ERES inhibition.

This review underscores the importance of developing specific and discrete modulators of the ERES, that preferably can be targeted to specific tissues, in a reversible manner. This requirement is of course complex, and demonstrates the challenge in developing modulators of ER exit that may be suitable for therapeutic development. The recent discovery of specific inhibitors of ERES, Retro-2 and 4-PBA are proofs of concept that this can successfully be done. However, until now, only the strongest and most general inhibitors have been recognised and studied. With an increase in the sensitivity of assays available for determining protein interactions and the recent increase in understanding transient interactions between intrinsically disordered domains and phase separation (Stancheva et al., 2020), we are now in a strong position to identify subtle changes in interactions between ERES proteins caused by potential therapeutic molecules. Additionally, with the recent leaps forward in microscopy developments that allow us to understand ERES formation and function at high time and spatial resolution, we also have the capabilities to recognise subtle mechanistic and functional changes at ERES. Achieving subtle modulation of ERES function is the key to identifying novel compounds that can successfully inhibit ERES, providing tuneable therapeutic effects while avoiding changes at a global level that will cause off target and unwanted side effects.
